# A study of booster dose influenza vaccination responses compared to standard dose in lupus patients: an open-labeled, randomized controlled study

**DOI:** 10.1007/s10238-025-01639-6

**Published:** 2025-04-09

**Authors:** Sasicha Yingyounyong, Pintip Ngamjanyaporn, Prapaporn Pisitkun, Kobporn Boonnak, Thanitta Suangtamai, Supranee Thongpradit, Porpon Rotjanapan

**Affiliations:** 1 Department of Medicine, Faculty of Medicine Ramathibodi Hospital, Mahidol University, Bangkok, Thailand; 2 Division of Allergy, Immunology, and Rheumatology, Department of Medicine, Faculty of Medicine Ramathibodi Hospital, Mahidol University, Bangkok, Thailand; 3 Department of Immunology, Faculty of Medicine Siriraj Hospital, Mahidol University, Bangkok, Thailand; 4 Research Center, Faculty of Medicine Ramathibodi Hospital, Mahidol University, Bangkok, Thailand; 5 Division of Infectious Diseases, Department of Medicine, Faculty of Medicine Ramathibodi Hospital, Mahidol University, 270 Rama VI Road, Ratchatewi, Bangkok, 10400 Thailand

**Keywords:** Lupus, Booster dose influenza vaccine, Seroprotection, Seroconversion

## Abstract

Despite receiving an annual influenza vaccination, lupus patients showed a decline in immunological responses for various reasons. This study aimed to assess immune responses after booster- (BD) and standard-dose (SD) quadrivalent influenza vaccine and the adverse events and incidence of influenza infection among lupus patients. A randomized controlled trial was conducted between March 2021 and May 2022 at Ramathibodi Hospital. All lupus patients were stratified into two groups depending on the depth of immunosuppressive therapy and randomized to receive either BD or SD. Hemagglutination inhibition assay (HAI) before vaccination and 4 weeks after completion of the vaccination series were assessed. The incidence of influenza infection and vaccine-associated adverse events were recorded. A total of 109 lupus patients completed the HAI analysis. 54/109 patients were in high- (HI), and 55/109 were in low-level immunosuppressive (LI) groups. Focusing at the rates to achieve HAI ≥ 1:160, in the LI group, the rates after SD were 85.5% for H1N1, 69.2% for H3N2/Hongkong, 82.8% for H3N2/Cambodia, 85.5% for B/Victoria, and 81.8% for B/Yamagata. After BD, the HAI titer rates of ≥ 1:160 cut point were increased in all strains, approaching 100%, similar to the HI group. There was one documented influenza infection during the 12-month follow-up period in LI who received SD. No serious adverse events associated with influenza vaccination were recorded. A booster dose of influenza vaccination may provide a higher HAI titer among lupus patients. The booster influenza vaccine regimen was considered safe in the BD group. Thai Clinical Trials Registry: TCTR20230610003.

**Supplementary Information:**

The online version contains supplementary material available at 10.1007/s10238-025-01639-6.

## Introduction

Influenza has been the leading cause of respiratory infection, with the highest positive rates in Central America, the Caribbean, Northern Africa, South-West Europe, and South-East Asia [1, 2]. In Thailand, Influenza annually presents two peaks: a major peak during the rainy season (June–August) and a minor peak in winter (October–February) [3]. The disease severity ranges from minor respiratory tract symptoms to severe pneumonia [4]. High rates of complications and hospitalization occur mainly in elders, patients with significant comorbidities, and those who received immunosuppressive agents [5].

In recent times, the efficacy of antiviral therapy has been proven by many studies on favorable clinical outcomes [6, 7]. Despite effective antiviral agents, the World Health Organization (WHO) estimates annual influenza epidemics include approximately 3–5 million cases of severe illness and 300,000–500,000 deaths [8]. Thus, influenza accounts for a large economic burden and psychologically affects society and the health care system [9, 10]. Therefore, disease prevention via immunization has been suggested to prevent detrimental consequences.

Hemagglutination inhibition assay (HAI) titers are used as surrogate markers of immune response and protection against influenza. For interpretation, seroprotection and seroconversion are often used to describe the susceptibility of a population to a specific strain of the virus [11]. According to the 2023 recommended adult and elderly immunization schedule from the Infectious Disease Association of Thailand, the annual conventional inactivated influenza vaccine during April–May is sufficient to prevent influenza infection and minimize the severity of the disease in the general public [11–14]. However, several studies have reported decreased antibody response in high-risk patients, including lupus. Dysregulated immune systems in these populations and the deterioration of CD4 T cells over time may lead to compromised vaccine-conferred protection [15–17]. Multiple strategies have been developed to increase antibody responses, such as intradermal administration, increasing antigen dosage in the vaccine, or administering booster doses [18, 19]. In 2017, Thomas et al. conducted a double-blind, multicenter, randomized controlled trial comparing 2 doses of high-dose trivalent to 2 doses of standard-dose quadrivalent influenza vaccine in allogeneic hematopoietic cell transplant adults. The study found that a 2-dose regimen of high-dose influenza vaccine was associated with greater immunogenicity and higher titers than the standard dose [20]. Although there are vaccine recommendations for those with immunosenescence, a consensus on the optimal immunization strategy for lupus patients has yet to be established. Limitations in most studies include interference by previous influenza vaccinations and differences in levels of individual immunosuppressive regimens, leading to the inaccuracy of HAI interpretation.

Therefore, we conducted a randomized controlled trial among Thais diagnosed with lupus to assess influenza vaccine response following two different regimens in Thailand.

## Objectives

The primary objective was to assess immune responses between booster- (BD) and standard-dose (SD) influenza vaccines in lupus patients by HAI. The secondary objectives were to evaluate the incidence of influenza infection and the rate of adverse events after the injections using Common Terminology Criteria for Adverse Events (CTCAE) version 5.

## Methods

### Study design

A randomized parallel design, controlled trial was conducted between March 2021 and May 2022 at Ramathibodi Hospital.

### Study population

All outpatients with lupus aged 18–65 were screened for eligibility. The upper limit at 65 years of age was established to eliminate the possible confounding factor from immunosenescence [21]. Enrolled patients were stratified into two groups depending on the depth of immunosuppressive therapy received (high-level (HI) and low-level intensity (LI)) and computer-based randomization to receive either BD or SD in a 1:1 ratio. After informed consent, data on patient demographics and relevant information were retrieved, including the Systemic Lupus Erythematosus Disease Activity Index 2000 score (SLEDAI-2 K). At enrollment, each patient received 0.1 mL of diphtheria-tetanus vaccine (dT) intradermally for testing T-cell responses. Blood samples were obtained for absolute lymphocyte counts and CD4 before vaccination. All subjects received a 0.5-mL dose of quadrivalent inactivated influenza vaccine intramuscularly from the 2021 Southern strain, containing 15 mcg each of A/Victoria(H1N1), A/Hongkong(H3N2), B/Victoria, and B/Yamagata for individuals enrolled in 2021, and 2021–2022 Northern strain containing A/Victoria(H1N1), A/Cambodia(H3N2), B/Victoria and B/Yamagata for individuals enrolled in 2022. For the participants in the BD group, the second influenza vaccine injection was given four weeks following the first injection. The HAI assessment was performed at three different time points: at enrollment (T0), 4 weeks after the first injection (T1), and 4 weeks after the second injection (T2) for individuals who received BD. Adverse events were monitored throughout the study, and a hospital visit was made for direct evaluation, if necessary. A history of flu-like illness was obtained using a standard questionnaire at every hospital visit until 12 months after enrollment.

### Inclusion and exclusion criteria

All lupus patients diagnosed by the 2019 European League Against Rheumatism/American College of Rheumatology (EULAR/ACR) SLE Classification criteria and aged 18–65 who had been under the care of rheumatologists at the Rheumatology Clinic of Ramathibodi Hospital were screened for eligibility.

The individuals with the following conditions were excluded from the study:

- Pregnancy
- Active cancer
- Positive HIV status
- Prior history of organ/hematopoietic stem cell transplant
- Receipt of influenza vaccine or previous history of an influenza infection within six months
- Evidence of cytomegalovirus infection within three months
- Known history of severe allergic reaction to influenza vaccine

### Outcome assessment

#### Hemagglutination inhibition assay (HAI) analysis

Blood samples were obtained and serums were separated by centrifuging at 1,500 × g for 10 min and stored at -80 °C until analysis. HAI analysis was performed by adding 300 µL of receptor-destroying enzyme to 25 µL serum and incubated at 37 °C overnight. Nonspecific inhibitors and agglutinators were removed by heat inactivation and adsorption with test red blood cells. The treated serum was then serially diluted, adding 8 HA units/50 mL of the virus antigen, which was received from the Department of Medical Sciences Ministry of Public Health, and incubating at 25 °C for 30 min. Hemagglutination inhibition was determined with 0.5% goose erythrocytes for H1N1 and influenza B and 0.75% guinea pig red blood cells for H3N2 at 25 °C for 30 min. The highest antibody dilution that inhibited hemagglutination was recorded as HAI titer.

#### Definitions

I *High- and low-intensity immunosuppressants* were defined and modified based on the Infectious Diseases Society of America Guidelines 2013 and Mycophenolate mofetil in the treatment of lupus nephritis by Patrick FK Yong and David P D’Cruz, as shown in Table 1 [22–24].

**Table 1:** Definition of high- and low-level immunosuppressive regimens [22–24]

Single drug regimen	
Low-level immunosuppressive	Prednisolone < 20 mg/day or equivalent for ≥ 14 days or alternate-day corticosteroid therapy
	Methotrexate ≤ 0.4 mg/kg/week
	Azathioprine ≤ 2 mg/kg/day
	6-mercaptopurine ≤ 1.5 mg/kg/day
	Mycophenolate mofetil ≤ 1.5 g/day
	Mycophenolate sodium ≤ 1,080 mg/day
	Immunosuppressive regimens other than those listed in high-level immunosuppression
High-level immunosuppressive	Prednisolone ≥ 20 mg/day or equivalent for ≥ 14 days
	Methotrexate > 0.4 mg/kg/week
	Azathioprine > 2 mg/kg/day
	6-mercaptopurine > 1.5 mg/kg/day
	Mycophenolate mofetil > 1.5 g/day
	Mycophenolate sodium > 1,080 mg/day
	Biologic immune modulators include tumor necrosis factor-alpha (TNF-α) blockers and rituximab
Combination regimen	
Low-level immunosuppressive	Combined two immunosuppressive drugs include methotrexate, azathioprine, 6-mercaptopurine, mycophenolate mofetil, and mycophenolate sodium in low-level immunosuppressive therapy definition
	Combined prednisolone with one of the immunosuppressive drugs includes methotrexate, azathioprine, 6-mercaptopurine, mycophenolate mofetil, and mycophenolate sodium in low-level immunosuppressive therapy definition
High-level immunosuppressive	Combined three or more drugs regimen include prednisolone or immunosuppressive drugs including methotrexate, azathioprine, 6-mercaptopurine, mycophenolate mofetil, and mycophenolate sodium at any dose
II *Influenza infection* was classified into three groups: Confirmed influenza infection, Influenza-like illness, and Acute respiratory tract infection. The diagnosis required clinical and laboratory criteria.
  1. Confirmed influenza infection: positive test from one of the following methods.
    Rapid Influenza Diagnostic Test (RIDT).
    Molecular diagnostic test.
    Viral isolation.
  Plus, flu-like symptoms within 10 days.
  Body temperature ≥ 38 °C and.
  One or more signs and symptoms of acute respiratory illness, e.g., cough, sore throat, nasal congestion, runny nose.
2. Influenza-like illness: symptoms within 10 days.
  Body temperature $\ge$ 38 °C and.
  One or more signs and symptoms of acute respiratory illness, e.g., cough, sore throat, nasal congestion, runny nose.
  Plus, risk factors such as close contact with influenza-infected patients or epidemic season of influenza.
3. Acute respiratory tract infection: symptoms within 10 days.
  Body temperature $\ge$ 38 °C or one or more signs and symptoms of acute respiratory illness, e.g., cough, sore throat, nasal congestion, runny nose.
III *Antibody response to influenza*: According to the WHO, seroprotection was defined as an HAI titer ≥ 1:40 based on a 50% reduction in disease; seroconversion was defined as more than a fourfold titer rise or more with the achievement of seroprotective titer [25, 26]. Titer < 1:10 were assigned a value of five for analysis.
IV *Active cancer status*: was defined as per Kearon C et al. [27].

#### Tetanus skin test interpretation

Inspection of the volar surface of the forearm at the injection site and skin indurations were measured at 48 h. A diameter of 5 mm or greater was defined as positive [28].

### Statistical analysis

Data were analyzed using STATA version 17.0. General characteristics of participants between groups were compared using Chi-square for categorical data, Mann–Whitney U test, and student’s t-test for continuous data. Linear mixed-effect models were used to evaluate the linear prediction of HAI at T0, T1, and T2. *P* values < 0.05 were considered statistical significance.

Sample sizes were calculated using the previous proportion of antibody response from Holvast A et al. and the formula to compare two independent proportions between HAI proportion after standard and HAI after booster dose vaccination [29]. Type 1 errors were replaced with 0.05, and Type 2 errors were with 0.2. Drop-out rates were calculated at 20%; hence, 128 samples can reject the null hypothesis.

## Results

### Patient characteristics

**Fig. 1:**
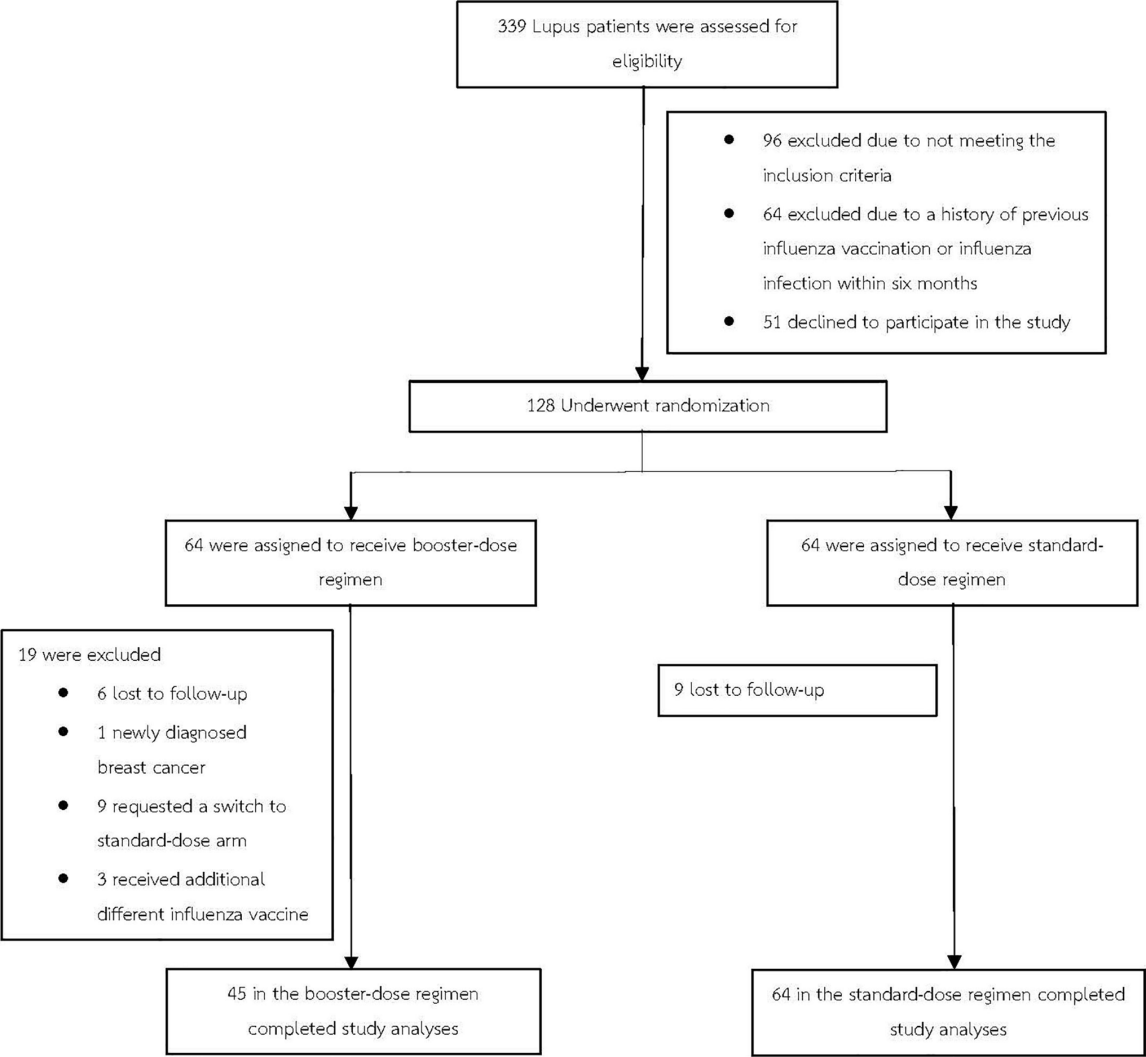
Protocol flow chart

Three hundred and thirty-nine patients were screened for eligibility, and 128 participants were enrolled and stratified into HI and LI groups. After randomization and follow-up, 45 patients in the BD group and 64 in the SD group completed the study protocol, as demonstrated in Fig. 1.

Baseline characteristics are demonstrated in Tables 2 and 3. The majority of the patients were female in all groups. Most patients had a SLEDAI-2 K score of four at enrollment and had no statistical difference between groups. Individuals in the HI group received up to five different immunosuppressive agents, while the LI group received a maximum of two agents. The most prescribed immunosuppressive agent among all participants was prednisolone.

**Table 2:** Baseline characteristics in low-intensity immunosuppressive group

	Low immunosuppressive		
	Standard dose, n = 33	Booster dose, n = 22	*p*-value
Age, mean ± SD., years	38.5 ± 7.8	37.8 ± 8.5	0.772
Sex, female, n (%)	31 (93.9)	21 (95.5)	1.000
Duration Dx SLE, median, years (range)	11.4 (5.9–20.2)	10.4 (4.1–14.2)	0.757
BMI, mean ± SD., Kg/m^2^	23.6 ± 4.0	24.7 ± 6.9	0.482
ALC, mean ± SD., cells/mm^3^	1363 ± 607	1616 ± 639	0.144
CD4, median (range), cells/mm^3^	450 (284–714)	418 (342–587)	0.929
SLEDAI, $\le$ 4, n (%)	33 (100)	20 (90.9)	0.156
dT skin test, positive, n (%)	26 (78.8)	20 (90.9)	0.289
Comorbidity, n (%)			
DM	1 (3.0)	1 (4.6)	1.000
CVS	5 (15.2)	1 (4.6)	0.384
CKD	2 (6.1)	1 (4.6)	1.000
Immunosuppressive, n (%)			0.311
none	2 (6.1)	0 (0)	
1 drug	12 (36.4)	5 (22.7)	
2 drugs	19 (57.6)	17 (77.3)	

*dT* diptheria tetanus vaccine, *SLE* systemic lupus erythematosus, *BMI* body mass index, *ALC* absolute lymphocyte count, *DM* diabetes mellitus, *CVS* cardiovascular syndrome, *CKD* chronic kidney disease

**Table 3:** Baseline characteristics in high-intensity immunosuppressive group

	High immunosuppressive		
	Standard dose, n = 31	Booster dose, n = 23	*p*-value
Age, mean ± SD., years	34.4 ± 9.5	32.1 ± 8.7	0.932
Sex, female, n (%)	28 (90.3)	23 (100)	0.253
Duration Dx SLE, median, years (range)	9.7 (5.6–17.3)	9.0 (3.5–12.7)	0.336
BMI, mean ± SD., Kg/m^2^	24.8 ± 4.8	23.9 ± 5.8	0.570
ALC, median (range), cells/mm^3^	1402 (918–2151)	1379 (871–2019)	0.630
CD4, median (range), cells/mm^3^	283 (119–559)	320 (179–483)	0.822
SLEDAI, $\le$ 4, n (%)	26 (83.9)	18 (78.3)	0.728
dT, positive, n (%)	21 (67.7)	16 (69.6)	0.887
Comorbidity, n (%)			
DM	1 (3.2)	0 (0)	1.000
CVS	3 (9.7)	1 (4.4)	0.628
CKD	3 (6.7)	1 (4.4)	0.628
Immunosuppressive, n (%)			0.942
1 drug	1 (3.2)	1 (4.4)	
2 drugs	7 (22.6)	7 (30.4)	
3 drugs	21 (67.7)	13 (56.5)	
4 drugs	1 (3.2)	1 (4.4)	
5 drugs	1 (3.2)	1 (4.4)	

*dT* Diptheria tetanus vaccine, *SLE* systemic lupus erythematosus, *BMI* body mass index, *ALC* absolute lymphocyte count, *DM* diabetes mellitus, *CVS* cardiovascular syndrome, *CKD* chronic kidney disease

At baseline, tetanus skin tests (TST) were found negative in 9/55 (16.4%) patients in the LI group and 17/54 (31.5%) in the HI group with no statistical significance (*P* = 0.064).

### Seroprotection

In the HI group, the seroprotection rate, which is defined as an HAI titer of 1:40 or higher, at T0 did not differ between SD and BD in H1N1 (71% vs. 78.3%) and B/Vic strain (74.2% vs. 78.3%). A similar relationship was found in the LI group in H1N1 (78.8% vs. 72.7%) and H3N2/Hongkong (82.4% vs. 88.9%).

In the LI group, the overall seroprotection rates at T0 for each influenza strain were 76.4% in H1N1, 84.6% in H3N2/Hongkong, 93.1% in H3N2/Cambodia, 74.5% in B/Victoria, and 89.1% in B/Yamagata. In SD group, seroprotection rates at T1 were as follows: 93.9% in H1N1, 100% in H3N2/Hongkong, 100% in H3N2/Cambodia, 96.9% in B/Victoria, and 93.9% in B/Yamagata. In the BD group, seroprotection rates at T1 were as follows: 90.9% in H1N1, 100% in H3N2/Hongkong, 100% in H3N2/Cambodia, 90.9% in B/Victoria, and 100% in B/Yamagata. However, no additional patients achieved seroprotection at T2 in the BD group.

**Fig. 2:**
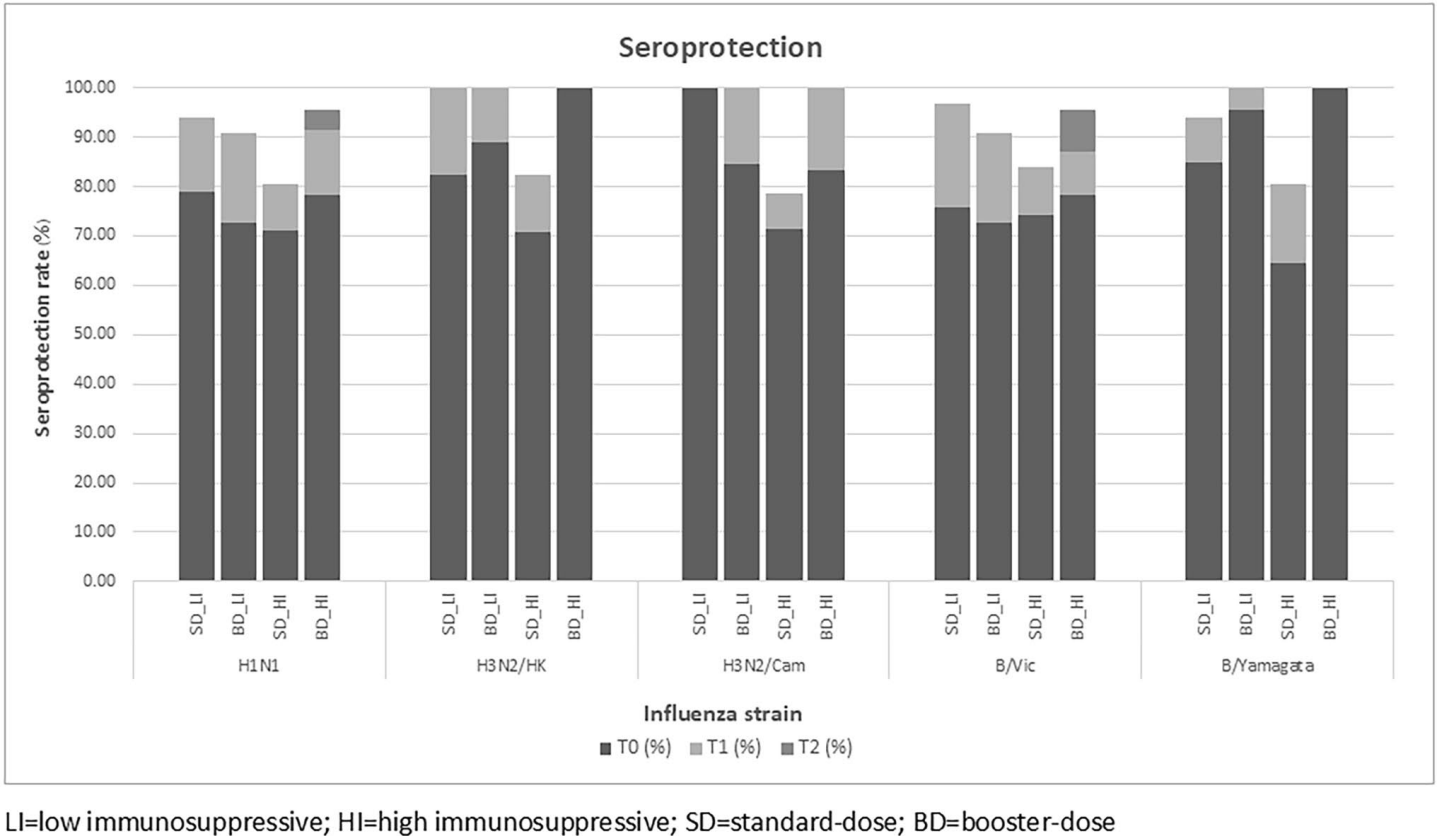
displays seroprotection rates with HAI titer ≥ 1:40 in low- and high-level immunosuppressive groups

In the HI group, similar seroprotection patterns were found in H3N2/Hongkong, H3N2/Cambodia, and B/Yamagata. For H1N1 and B/Victoria, the seroprotection rates at T1 and T2 time points were increased with no statistical significance in the BD group. For H1N1, the seroprotection rates are 78.3% at T0, 91.3% at T1, and 95.6% at T2 (*P* = 0.980). Seroprotection rates in B/Victoria are 78.3, 86.9, and 95.6%, respectively (*P* = 0.500) (Fig. 2) (Supplementary Table S1).

### Seroconversion

In the LI group, seroconversion at T1 was found in all influenza strains. When using baseline titer as a reference, the seroconversion rates at the T2 time point were higher than T1 in H3N2/Hongkong (68.2% vs. 95.5%, *P* = 0.031). In other strains, some additional patients had seroconversion at T2 with no statistical significance, including H1N1 (54.6% vs. 68.2%, *P* = 0.250), B/Victoria (54.6% vs. 59.1%, *P* = 1.000) and B/Yamagata (31.8% vs. 54.6%, *P* = 0.125). In contrast, no further seroconversion was achieved at the T2 time point in H3N2/Cambodia.

**Fig. 3:**
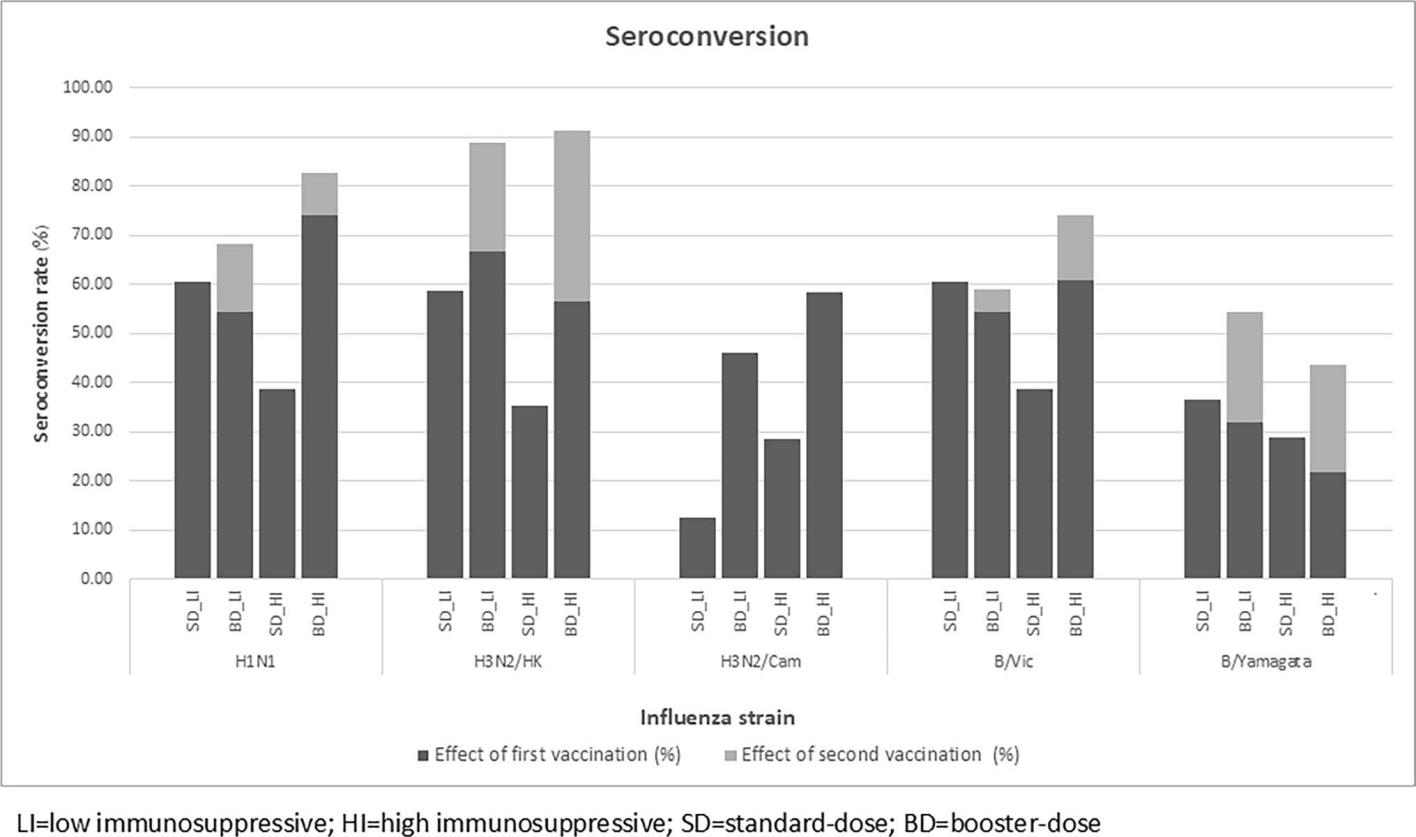
displays seroconversion rates in low- and in high-level immunosuppressive groups

Similar outcomes were found in the HI group; seroconversion was found in all strains at the T1 time point. At T2, there was a significant increase in seroconversion rates in H3N2/Hongkong (56.5% vs. 91.3%, *P* = 0.008) and an increase in seroconversion rate with no statistical significance in other strains, including H1N1 (73.9% vs. 82.6%, *P* = 0.500) and B/Victoria (60.9% vs. 73.9%, *P* = 0.250) B/Yamagata (21.7% vs. 43.5%, *P* = 0.125) (Fig. 3) (Supplementary table S2).

There was no statistically significant association between TST and seroconversion in all influenza strains in both HI and LI groups (p value > 0.05).

### Hemagglutination inhibition titer ≥ 1:160

The analysis of the proportion of lupus patients with HAI ≥ 1:160, aiming to protect up to 95% of patients against influenza infection, was performed and compared between the BD- and SD-vaccination groups [30]. In the LI group, the rate of HAI ≥ 1:160 titer after SD was 85.5% in H1N1, 69.2% in H3N2/Hongkong, 82.8% in H3N2/Cambodia, 85.5% in B/Victoria and 81.8% in B/Yamagata. While after the BD, the rates of HAI ≥ 1:160 titer were 90.9% in H1N1, 100% in H3N2/Hongkong, 100% in H3N2/Cambodia, 90.9% in B/Victoria, and 90.9% in B/Yamagata.

**Fig. 4:**
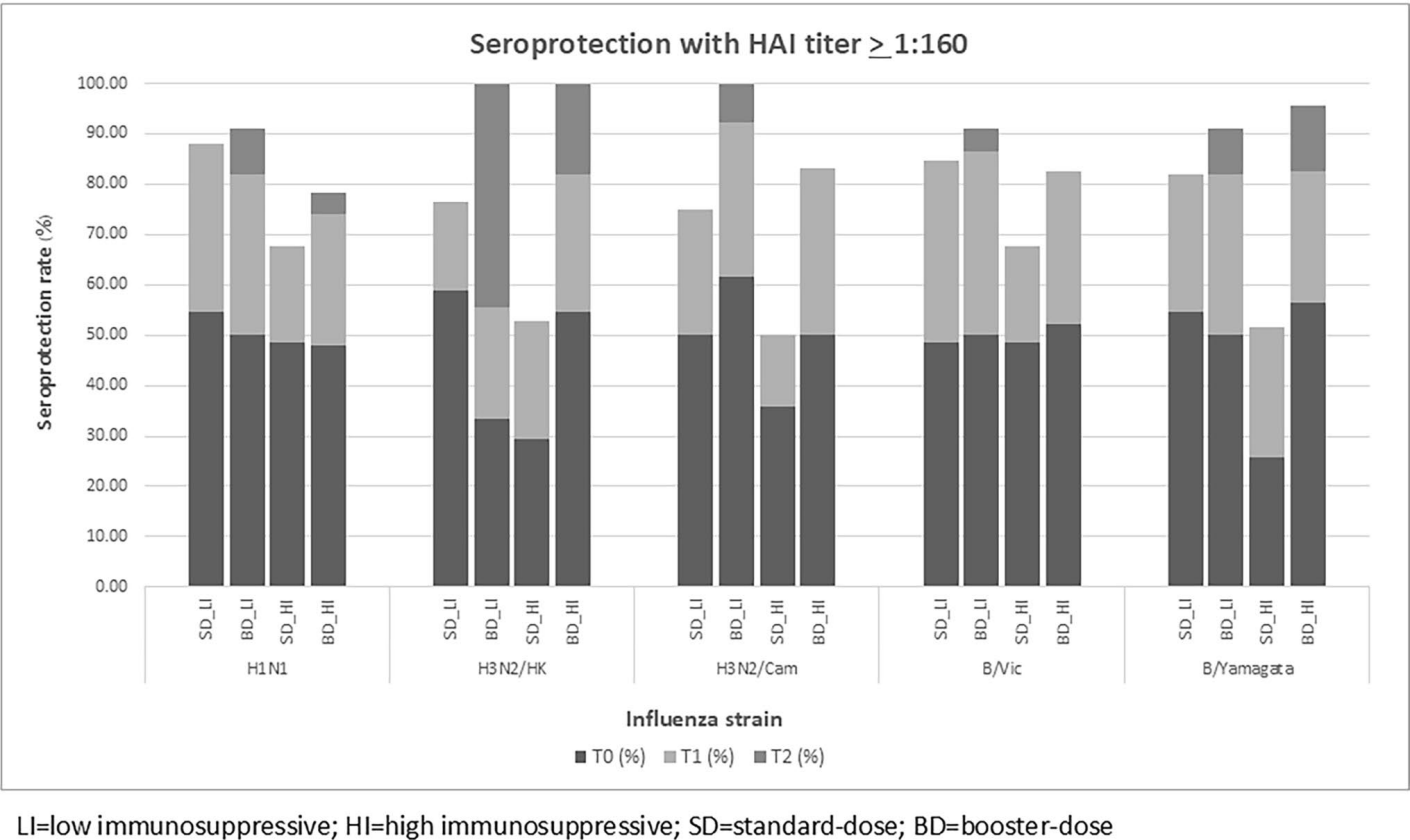
displays seroprotection rates with HAI titer ≥ 1:160 in low- and in high-level immunosuppressive groups

Similar results were found in the HI group, with the rate of HAI ≥ 1:160 titer after the SD was 70.4% in H1N1, 64.3% in H3N2/Hongkong, 65.4% in H3N2/Cambodia, 74.1% in B/Victoria and 64.8% in B/Yamagata after BD were 78.3% in H1N1, 100% in H3N2/Hongkong, 83.3% in H3N2/Cambodia, 78.3% in B/Victoria and 95.7% in B/Yamagata (Fig. 4). The difference was found only in B/Yamagata with *P* = 0.013.

### Mixed-effect model analysis

The linear mixed-effects models utilized unadjusted data from HAI to analyze the temporal trend of the raw mean HAI titer. This approach was necessitated by the constraints in evaluating seroprotection and seroconversion, which were restricted to a threshold of 1:40 for the HAI titer. The mean HAI titer in each influenza strain was plotted on the Y-axis; the X-axis represents sampling time. We compared the mean HAI titer level between SD and BD groups at different time frames to evaluate the evolution of the titer (Supplementary Figs. S1–S5).

In H1N1, the mean HAI titer in SD was approximated to the BD group in both LI and HI groups at baseline. After the second vaccination, the mean HAI titer in the HI group increased from 1555.6 to 2146.5, while in the LI group, the titer did not change (1302.3 to 1287.9).

Similar results were found in B/Victoria. The mean HAI titer in the HI group increased after the second vaccination (1874.1 to 2134.1 units), while the mean titer in LI slightly decreased (1422.3 to 1397.0 units).

In H3N2/Hongkong, HAI titer trends were similar in LI and HI groups. After the second vaccination, HAI titers in the HI group increased from 187.8 to 681.7 units and 107.3 to 407.3 units in the LI group. Similar trends were found in B/Yamagata. HAI titer increased after the second vaccination in both HI and LI groups (431.3 to 921.7 in the HI group and 710.9 to 1030 in the LI group).

### Rate of influenza infection and adverse events

Confirmed influenza infection was documented in one patient who received SD and was in the LI group. Influenza-like illness was found in 19/109(17.4%) patients; among these, 10/19 were in the SD group, and acute respiratory tract infection was found in 37(33.9%) patients, and 19/37 among them were in the SD group. None of the participants were hospitalized with severe influenza virus pneumonia or influenza-related reasons.

The study found no systemic adverse events in any patients. Grade 1 injection site reactions were found in 19/109(17.4%) lupus patients, and there was no difference between SD and BD.

## Discussion

Influenza immunization is the primary strategy for preventing and reducing complications from the infection. In high-risk groups such as the elderly, children, pregnant and postpartum individuals, the influenza vaccine has been documented to be a cost-effective prescription from a pharmacoeconomic point of view [31–33]. However, the knowledge regarding vaccine efficacy and effectiveness in special populations, particularly immunocompromised patients receiving immunosuppressive therapy, is scarce.

Influenza infection increases morbidity and mortality in immunocompromised patients in general, possibly including lupus as well despite the current vaccination scheme due to lower immunological responses [34]. Relevant factors hypothesized to explain this phenomenon include immunosuppressants as a patient factor, inappropriate doses, and adjuvant influenza antigen as modifiable vaccine factors [35–37]. The high-dose quadrivalent influenza vaccine has been advocated in older populations to enhance antibody response following vaccination. Still, no recommendation has been made for immunocompromised patients with lupus yet.

In this study, we evaluated the impact of the BD regimen and the potential effect of immunosuppressive therapy on antibody production following influenza vaccination. A Holvast A. et al. study revealed that seroconversion rates were higher in patients who received a booster dose of influenza vaccine. Still, no significant changes in seroprotection were documented. The potential explanation was due to the effect of the vaccination in the previous year [29].

The current study enrolled participants mostly in their young age as the mean ages in all groups were under 40 years. Therefore, the possibility of having poorer vaccine immune responses from chronological age can be discarded. At baseline, more than 70% of the HI and LI patients already had seroprotection in all influenza strains, which may represent their baseline immune responses were relatively good, consistent with TST results. After the first vaccination, seroprotection rates were increased in all strains. Nevertheless, no additional seroprotection was found in the LI group after the second vaccination. In the HI group, increases in seroprotection rates were found only in H1N1 and B/Victoria after the second vaccination. These results were consistent with a previous study in which a booster dose of vaccine can cause a slight increase in seroprotection rates against H1N1 and B strains [29]. The main reason that additional seroprotection after the second dose was observed in only the HI group may be that over 90% of the patients in the LI group already achieved seroprotection after the first injection. These findings may imply a relatively appropriate influenza vaccination response among most lupus patients receiving standard immunization schedules. However, to achieve seroprotection at titer ≥ 1:40, approximately 50% of the patients remain at risk of having influenza acquisition, and this might not be the most desirable outcome, particularly among immunocompromised patients. Based on a previous study, the HAI titer ≥ 1:160 provided more extensive protection from an individual to acquire the infection up to at least 90% [30, 38]. This study found more patients who reached these titer levels in the BD group in the HI and LI groups. This finding indicated that lupus patients who received HI or LI benefited from the BD regimen to obtain the most desirable protection level from influenza vaccination.

Regarding the seroconversion rate aspect, a significant increase was observed in the H3N2/Hongkong strain after the second vaccination, both in HI and LI groups but not in other strains. The plausible explanation that seroconversion rates were not significantly observed in most patients and strains was 1.) The HAI titers among several participants at T1 were already at high levels, and 2.) The limitation of the HAI laboratory study was that it did not allow us to learn the real titer, as the upper limit of antibody titer detection was 1:5120. Therefore, the actual seroconversion rates were not justified in this group of patients. An exception was identified in the seroconversion rates following the initial vaccination with the H3N2/Cambodia strain, observed in both the low-level group (12.5% in SD vs. 46.1% in BD) and the high-level groups (28.6% in SD vs. 58.3% in BD). The hypothesis to explain such a phenomenon was that the effects of repeated vaccinations in the prior years may influence the observed disparity in the baseline HAI titer and immune response. However, the information in this aspect was not collected [39].

Due to some limitations in seroprotection and seroconversion assessment, we used mixed model analysis to evaluate trends of mean HAI titer at T0, T1, and T2 compared between HI and LI groups. The findings revealed that the second vaccination induced higher HAI titer against H3N2/Hongkong and B/Yamagata regardless of the depth of immunosuppressive levels.

In this study, reported influenza cases were much less than in prior years. According to the Global overview of influenza circulation from late 2019 to 2020 by Karlsson et al., the influenza infection rate markedly decreased during the COVID-19 pandemic. Our study confirmed that the influenza infection rate was lower (0.9%) than global data (19%) [40]. None of the patients was documented with influenza pneumonia or hospitalization from influenza infection. None of the patients in the standard and booster dose groups developed a documented disease flare. Wearing face masks and keeping their distance from one another were common practices among Thais during the COVID-19 pandemic, which could explain the lower influenza infection rates among participants in this study.

Regarding safety concerns, the BD did not increase adverse events or lupus disease activity during the study period. The major strength of this study was that it stratified baseline patients with the depth of immunosuppressive therapy, considered an essential variable that may affect immunogenicity, representing a real-world setting. However, the study has some limitations: (1) Deviation of HAI assessment from the original protocol in some participants due to the COVID-19 pandemic that did not allow scheduled hospital visits to occur as planned, mainly in the BD group, (2) The study period involved two calendar years. Therefore, different H3N2 strains were used for analysis, (3) Relatively diverse follow-up visit intervals from 4 to 12 weeks in some participants despite being the minority, as the impact of the COVID-19 pandemic might have lowered the accuracy of the HAI titer interpretation, (4) Lack of cell-mediated immune response assessment following influenza vaccination, (5) The common practice of wearing face masks among Thais during the COVID-19 pandemic may have skewed the true incidence of influenza during the study period, and (6) A cost-effectiveness analysis was not performed in this manuscript.

According to the Centers for Medicare & Medicaid Services of the United States, a high-dose influenza vaccine costs approximately $73.40 per dose. In contrast, the booster-dose vaccination used in this study was $15 per course, including tax [41]. Although Thailand is classified as an upper-middle-income country, universal prescribing of a high-dose influenza vaccine to individuals at risk is deemed inappropriate in several circumstances regardless of the vaccine approval indication for public use, and a booster dose of the standard vaccine may be a reasonable option.

## Conclusions

The current study showed that standard influenza vaccination provided a good immune response among lupus patients; however, approximately 25% of patients did not achieve a titer of ≥ 1:160. The BD regimen may result in better immune responses and increased seroconversion in some strains, especially in patients who received HI. However, there were no clinical differences in the incidence of influenza infection between the SD and BD regimens. Further studies on a larger scale are necessary.

## Supplementary Information

Below is the link to the electronic supplementary material.

Supplementary file1 (DOCX 937 kb)

Supplementary file2 (DOCX 107 kb)

## Data Availability

No datasets were generated or analyzed during the current study.
