# Supplementary figures and images for: A study of booster dose influenza vaccination responses compared to standard dose in lupus patients: an open-labeled, randomized controlled study
Source: Clin Exp Med. 2025 Apr 9;25(1):109. doi: 10.1007/s10238-025-01639-6 (PMC11982163; doi:10.1007/s10238-025-01639-6)

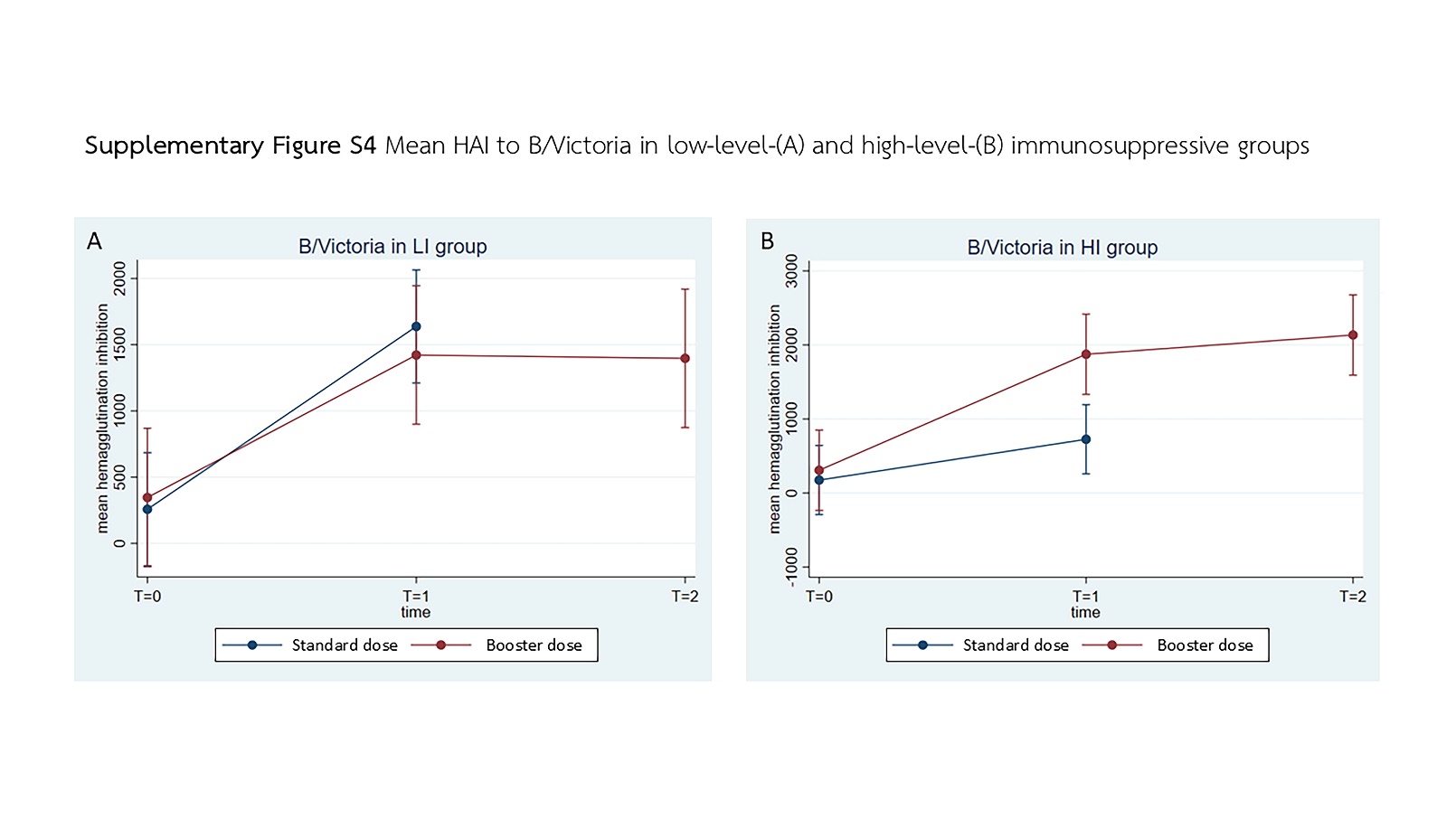

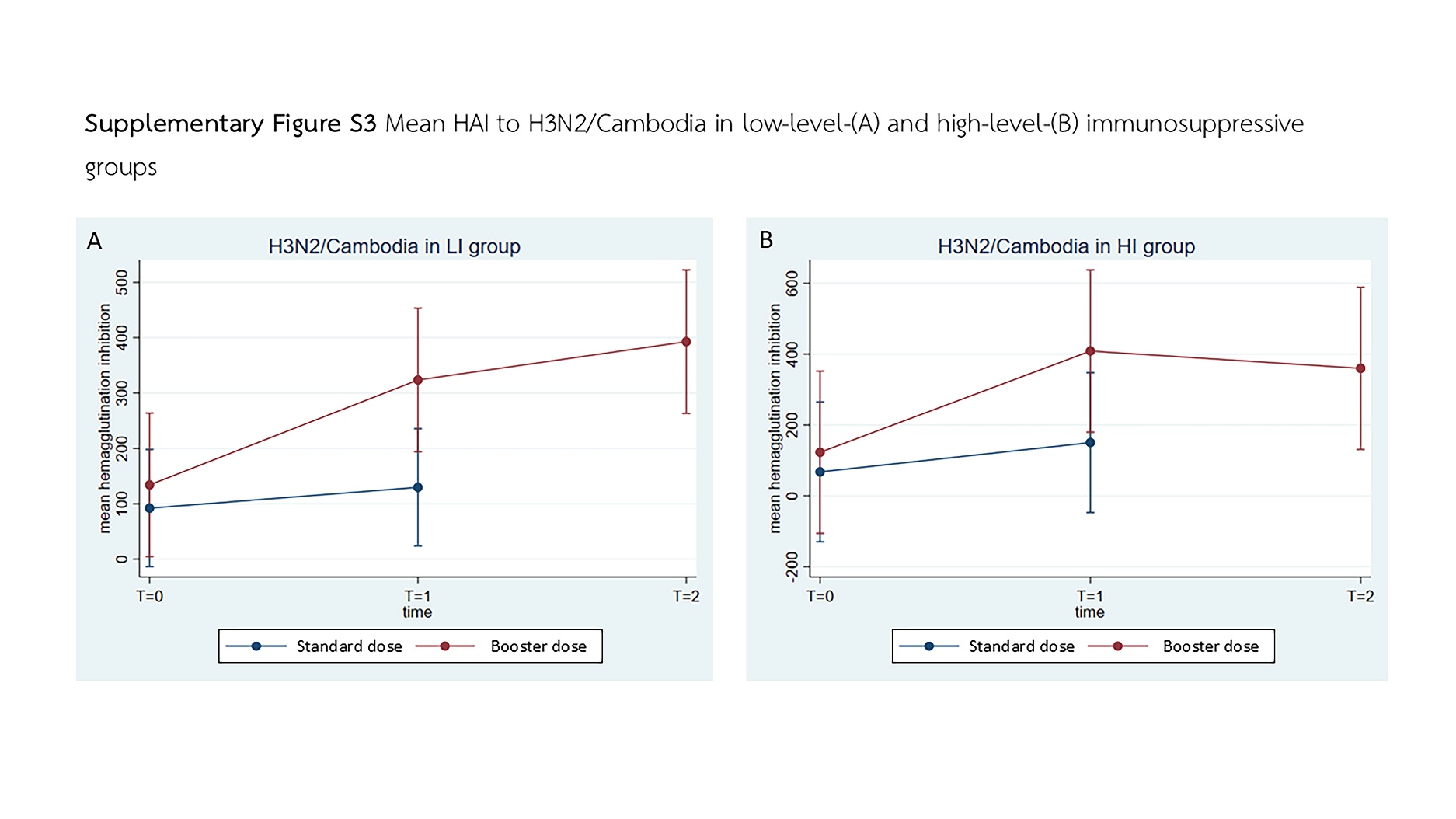

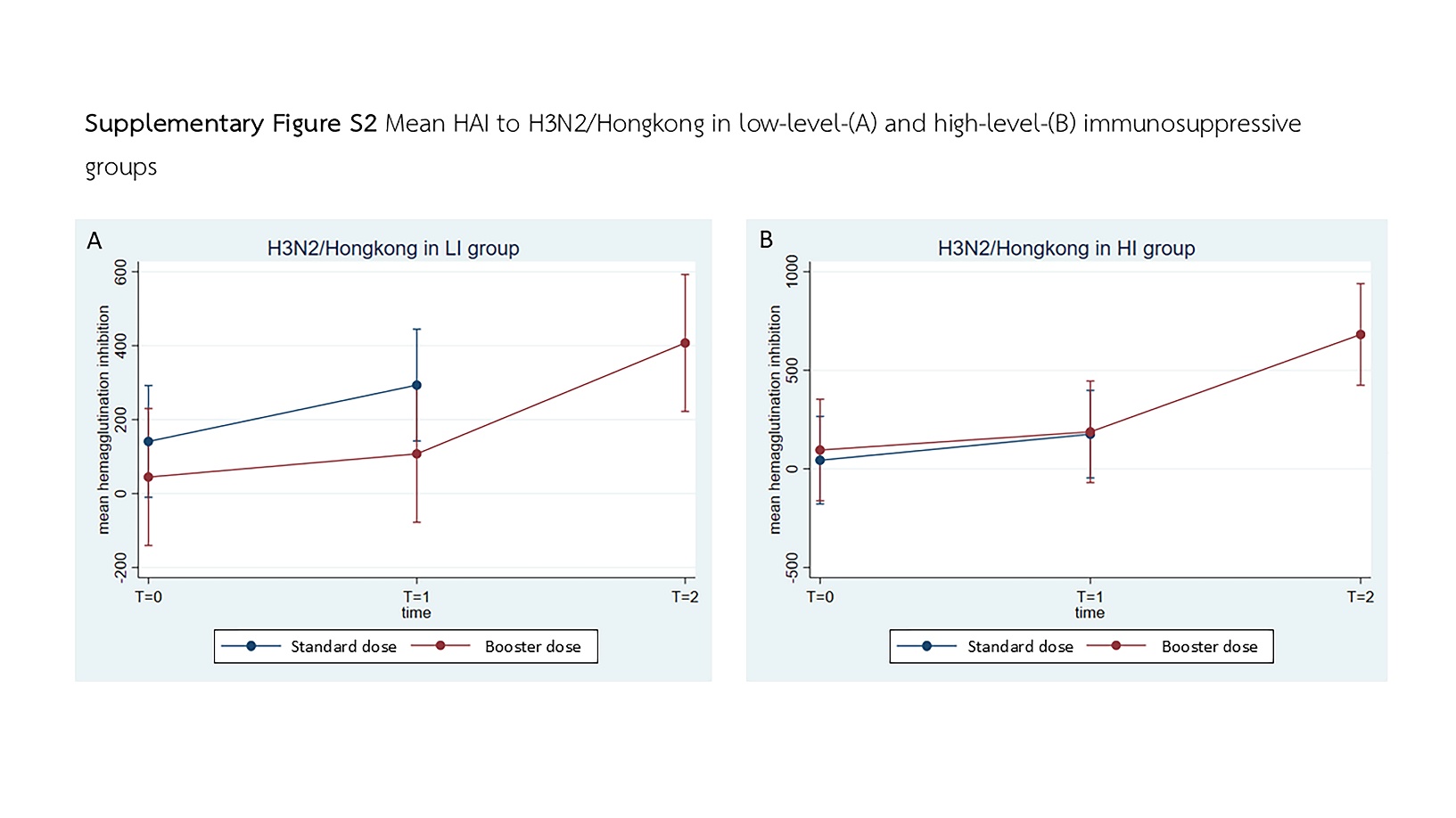

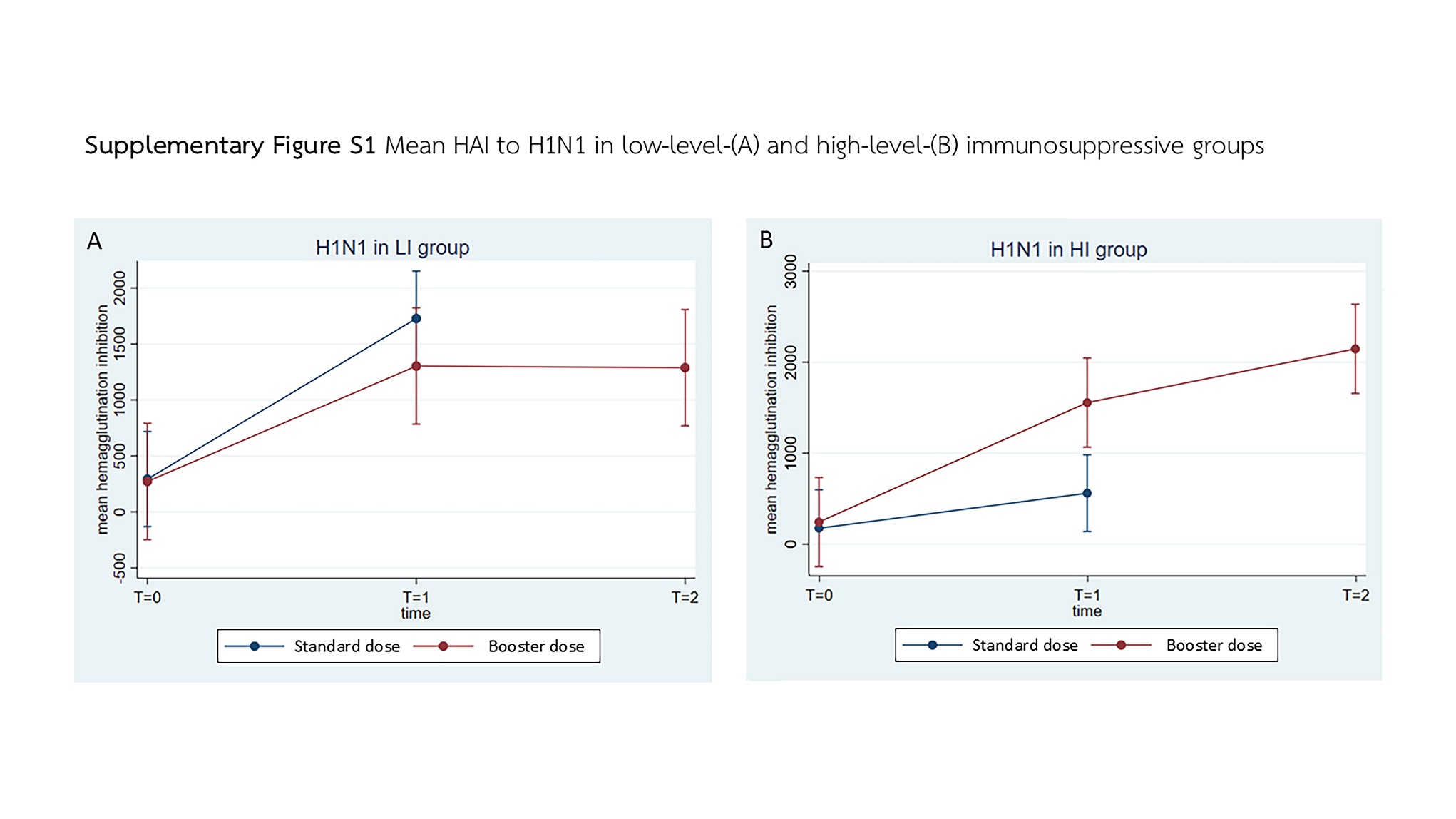

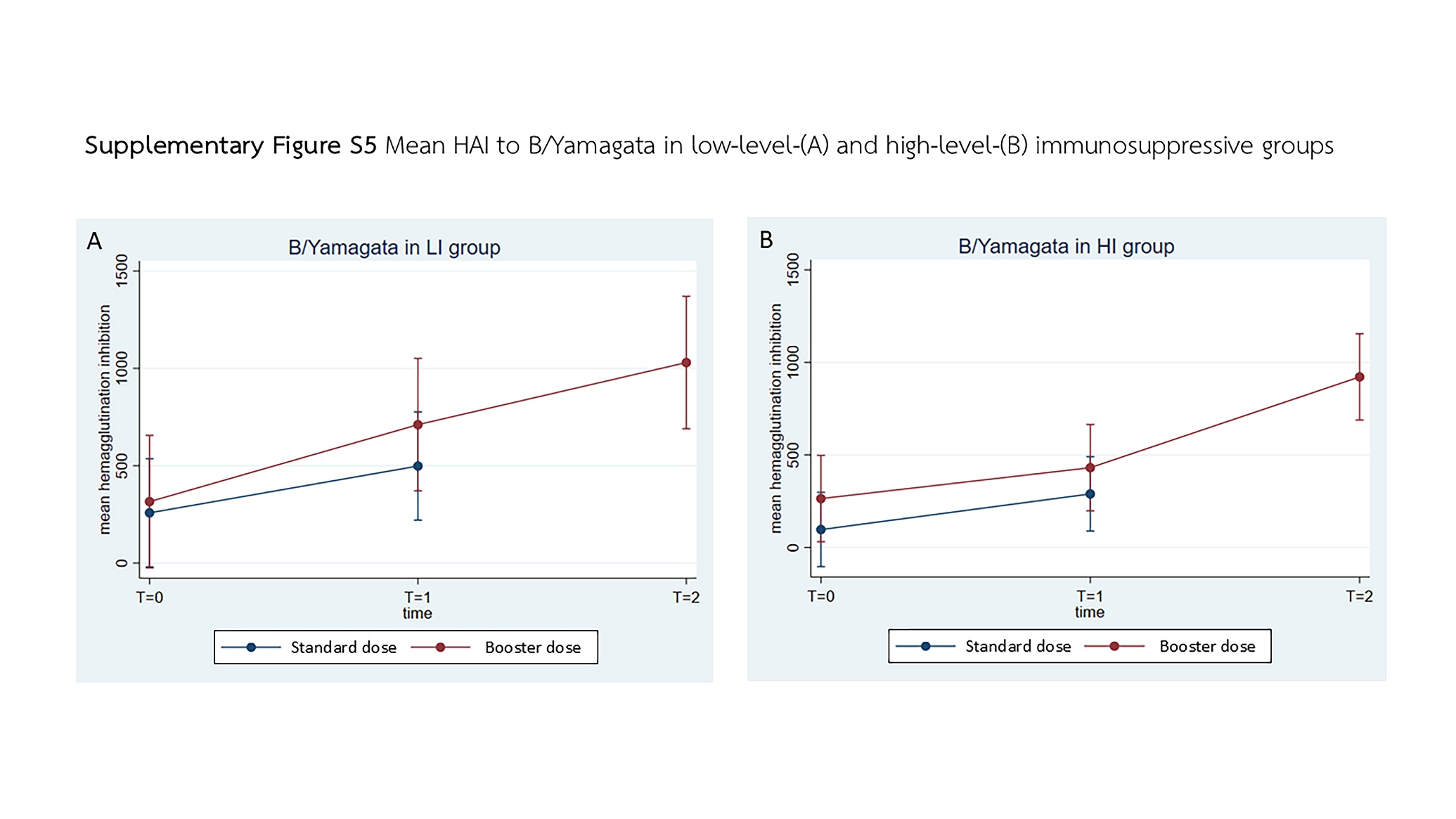

Supplement: Supplementary file 1 — Supplementary file1 (DOCX 937 kb) [file 10238_2025_1639_MOESM1_ESM.docx]
