# Supplementary material for: A study of booster dose influenza vaccination responses compared to standard dose in lupus patients: an open-labeled, randomized controlled study
Source: Clin Exp Med. 2025 Apr 9;25(1):109. doi: 10.1007/s10238-025-01639-6 (PMC11982163; doi:10.1007/s10238-025-01639-6)
Supplement: Supplementary file 2 [file 10238_2025_1639_MOESM2_ESM.docx]

**Supplementary Table S1** demonstrates the seroprotection rate in low- and high-level immunosupp

| | Low-level immunosuppressive | | | High-level immunosuppressiv | |
|---|---|---|---|---|---|
| Seroprotection rate in SD group, n (%) | | | | Seroprotection rate in SD group, n | |
| Influenza strain\Time | T0 | T1 | T2 | T0 | T1 |
| H1N1 | 26 (78.8) | 31 (93.9) | NA | 22 (71.0) | 25 (80.6) |
| H3N2/Hongkong | 14 (82.4) | 17 (100) | NA | 12 (70.6) | 14 (82.4) |
| H3N2/Cambodia | 16 (100) | 16 (100) | NA | 10 (71.4) | 11 (78.6) |
| B/Victoria | 25 (75.8) | 32 (96.9) | NA | 23 (74.2) | 26 (83.9) |
| B/Yamagata | 28 (84.8) | 31 (93.9) | NA | 20 (64.5) | 25 (80.6) |
| Seroprotection rate in BD group, n (%) | | | | Seroprotection rate in BD group, n | |
| H1N1 | 16 (72.7) | 20 (90.9) | 20 (90.9) | 18 (78.3) | 21 (91.3) |
| H3N2/Hongkong | 8 (88.9) | 9 (100) | 9 (100) | 11 (100) | 11 (100) |
| H3N2/Cambodia | 11 (84.6) | 13 (100) | 13 (100) | 10 (83.3) | 12 (100) |
| B/Victoria | 16 (72.7) | 20 (90.9) | 20 (90.9) | 18 (78.3) | 20 (86.9) |
| B/Yamagata | 21 (95.5) | 22 (100) | 22 (100) | 23 (100) | 23 (100) |

**Supplementary Table S2** demonstrates the seroconversion rate in low- and high-level immunosup

| | Low-level immunosuppressive | | High-level immunosu |
|---|---|---|---|
| Seroconversion rate in SD group, n (%) | | | Seroconversion rate in SD |
| Influenza strain\Time | After first vaccination | After second vaccination | After first vaccination |
| H1N1 | 20 (60.6) | NA | 12 (38.7) |
| H3N2/Hongkong | 10 (58.8) | NA | 6 (35.3) |
| H3N2/Cambodia | 2 (12.5) | NA | 4 (28.6) |
| B/Victoria | 22 (66.7) | NA | 12 (38.7) |
| B/Yamagata | 12 (36.4) | NA | 9 (29) |
| Seroconversion rate in BD group, n (%) | | | Seroconversion rate in BD |
| H1N1 | 12 (54.5) | 15 (68.2) | 17 (73.9) |
| H3N2/Hongkong | 6 (66.7) | 8 (88.9) | 6 (56.5) |
| H3N2/Cambodia | 6 (46.2) | 6 (46.2) | 7 (58.3) |
| B/Victoria | 12 (54.5) | 13 (59.1) | 14 (60.9) |
| B/Yamagata | 8 (36.4) | 12 (54.6) | 5 (21.7) |

SD: standard-dose; BD: booster-dose; T0: prior to vaccination; T1: 4 weeks after first vaccination; T2: 4 week vaccination; *P=0.008 (T2 vs T1).

**Supplementary Table S3** demonstrates geometric mean titer in low-level immunosuppressive gro[illegible]

| **Low-level immunosuppressive** | | | |
|---|---|---|---|
| GMT in SD group | | | |
| Influenza strain\Time | T0 | T1 | T2 |
| H1N1 | 112.0 | 789.6 | NA |
| H3N2/Hongkong | 110.9 | 347.2 | NA |
| H3N2/Cambodia | 128.8 | 198.7 | NA |
| B/Victoria | 100.7 | 741.4 | NA |
| B/Yamagata | 119.2 | 282.1 | NA |
| GMT in BD group | | | |
| H1N1 | 80.0 | 482.0 | 546.7 |
| H3N2/Hongkong | 80.0 | 172.8 | 640.0 |
| H3N2/Cambodia | 129.3 | 417.8 | 490.2 |
| B/Victoria | 96.6 | 546.7 | 640.0 |
| B/Yamagata | 150.2 | 351.7 | 513.3 |

GMT: geometric mean titer; SD: standard-dose; BD: booster-dose; T0: prior to vaccination; T1: 4 weeks after first vaccination; T2: 4 weeks after second vaccination

**Supplementary Table S4** demonstrates geometric mean titer in high-level immunosuppressive gr[illegible]

| **High-level immunosuppressive** | | | |
|---|---|---|---|
| GMT in SD group | | | |
| Influenza strain\Time | T0 | T1 | T2 |
| H1N1 | 68.4 | 187.1 | NA |
| H3N2/Hongkong | 55.4 | 125.3 | NA |
| H3N2/Cambodia | 65.6 | 131.3 | NA |
| B/Victoria | 66.9 | 239.3 | NA |
| B/Yamagata | 50.0 | 109.4 | NA |
| GMT in BD group | | | |
| H1N1 | 95.9 | 518.3 | 766.8 |
| H3N2/Hongkong | 132.4 | 264.9 | 726.0 |
